# Editorial: Innovations in dementia and ageing care

**DOI:** 10.3389/fresc.2023.1191633

**Published:** 2023-04-26

**Authors:** Catherine Quinn, Emma Wolverson, Gail Mountain

**Affiliations:** ^1^ Centre for Applied Dementia Studies, University of Bradford, Bradford, United Kingdom; ^2^ Wolfson Centre for Applied Health Research, Bradford, United Kingdom; ^3^ Faculty of Health Sciences, University of Hull, Hull, United Kingdom; ^4^ Dementia UK, London, United Kingdom

**Keywords:** Alzheimer's disease, carer, psychosocial interventions, health serivces, community, quality of life, frailty

Editorial on the Research Topic Innovations in dementia and ageing care

The COVID-19 pandemic has highlighted how quality of life can readily be compromised in vulnerable older people and the fragility of health and care systems that aim to promote well-being in this population (1, 2). People with dementia have been impacted by diminished face-to-face access to health, care and community services (2–4). Supporters of people with dementia were also significantly affected (5, 6). Many services have still not re-opened, funding revenues for the voluntary sector have been affected and there has been a shift to delivering services remotely either over the telephone or online, e.g., telemedicine or telehealth (7). The challenges faced during the height of the pandemic and this period afterwards also present opportunities to consider how health and social care services and community assets can be remodeled to be more responsive to the physical and psychosocial needs of this population. In this Research Topic we focused on innovative approaches in dementia and ageing care to identify ways in which people can be better supported. The papers selected highlight opportunities to make changes within hospitals, primary care, memory clinics, the community and also in the way services are developed. Although dementia is the focus of all the papers within this special edition the themes that run through them have relevance to a wider population of frail older people.

An important aspect of developing effective support for individuals with dementia is to understand what it means to them to live well (8). Increasingly people living with a diagnosis of dementia are seeking to share their experiences to improve the support and experiences of others. The paper by West et al. describes an innovative approach of involving people with dementia in co-producing and co-delivering a course within a mental health Recovery college. Recovery colleges are educational settings for people who access mental health services, their families and staff. The “process” of recovery within these colleges focuses on the core outcomes of connecting with others, inspiring hope, maintaining a positive identity, finding meaning and empowerment. This study found that this co-production approach could be a positive experience for all involved and offer opportunities for vicarious learning.

Recovery colleges offer peer-support and people with dementia and carers often value having opportunities to meet others in a similar situation to share their concerns and learn from others who would understand them (9). Peer support can be offered within other group-based interventions. The paper by Chadwick et al. evaluated an exercise programme for people with dementia and carers, identifying multiple benefits in terms of being able to take part in activities they enjoy and helping them to maintain their identity. The activities offered within this group offered an opportunity for social interaction and peer support. The paper identified the value of having trained facilitators to run this group to ensure meaningful inclusion.

In considering the effectiveness of support interventions it is necessary to reflect on how to evaluate such approaches. The paper by Smith et al. considers the value of participation as a construct in dementia research and care. It explores how we might conceptualise and measure participation and what value it might bring to evaluating interventions that aim to affect psychosocial health. Despite the fact that people with dementia have identified participation as a meaningful outcome for them, researchers often lack a unified definition of what participation means. This paper adds to the debate about how we capture the perspectives of people with dementia about what they consider as meaningful outcomes in evaluating research.

Providing effective support to individuals involves taking a personalised approach that recognises that individuals will have different needs. The person's psychological characteristics and health has been identified as an important determinant of living well with dementia (10). This considers both positive and negative emotional states. One psychological factor that can influence behaviours is trauma, and this a particularly pertinent topic in the current situation as long-term psychological impact of the pandemic is still unknown. Couzner et al. used a novel approach of trauma-informed care to identify whether past traumatic events might be having an impact on the behaviour of the person with dementia in a hospital ward. By identifying past traumatic events in the individuals lives this led to a change in the approach taken to care for them leading to improvements in their well-being. The paper by Moniz-Cook and Mountain also advocates for a more personalised psychosocial approach. Describing a study of a memory clinic primary care approach, this highlights the need for collaborative approaches to providing care to ensure better care co-ordination. Within this paper the authors reflect on the impact of the pandemic on services and identify that the already limited availability of post-diagnostic support was further affected due to the pandemic.

The COVID-19 pandemic has highlighted the need for innovative approaches in treatment through health services as well as support through community and social services. However, we need to also ensure that innovation is relevant and accessible. On-line provision is one form of innovation that has proliferated. However, this has disenfranchised many; for example, older people may be at more risk of digital exclusion (11). We recommend that there should be a holistic approach towards assessing the needs of people, and subsequent services should be tailored to meet those needs. Interventions should seek to enhance identity, find meaning, promote empowerment and foster opportunities for peer support. Involving older people and people with dementia directly in the development of new interventions and services is one way of trying to ensure that services meet needs.

## References

[1] Age UK. The impact of Covid-19 to date on older people’s mental and physical health. Age UK (2020).

[2] Alzheimer's Society. Worst hit: dementia during coronavirus report. London (2020).

[3] van Horik JO Collins R Martyr A Henderson C Jones RW Knapp M Limited receipt of support services among people with mild-to-moderate dementia: findings from the ideal cohort. Int J Geriatr Psychiatry. (2022) 37(3). 10.1002/gps.5688 35128725 PMC9306706

[4] Clare L Martyr A Gamble LD Pentecost C Collins R Dawson E Impact of COVID-19 on ‘living well’ with mild-to-moderate dementia in the community: findings from the ideal cohort. J Alzheimers Dis. (2022) 85(2):925–40. 10.3233/JAD-215095 34776448

[5] Quinn C Gamble LD Parker S Martyr A Collins R Victor C Impact of COVID-19 on carers of people with dementia in the community: findings from the British ideal cohort. Int J Geriatr Psychiatry. (2022) 37(5). 10.1002/gps.5708 PMC9087398 35394090

[6] Sriram V Jenkinson C Peters M. Impact of COVID-19 restrictions on carers of persons with dementia in the UK: a qualitative study. Age Ageing. (2021) 50(6):1876–85. 10.1093/ageing/afab156 34224555 PMC8384409

[7] Liu KY Howard R Banerjee S Comas-Herrera A Goddard J Knapp M Dementia wellbeing and COVID-19: review and expert consensus on current research and knowledge gaps. Int J Geriatr Psychiatry. (2021) 36(11):1597–639. 10.1002/gps.5567 34043836 PMC8237017

[8] Quinn C Pickett JA Litherland R Morris RG Martyr A Clare L Living well with dementia: what is possible and how to promote it. Int J Geriatr Psychiatry. (2022) 37(1). 10.1002/gps.5627 PMC9292841 34564897

[9] Quinn C Hart N Henderson C Litherland R Pickett J Clare L. Developing supportive local communities: perspectives from people with dementia and caregivers participating in the ideal programme. J Aging Soc Policy. (2022) 34(6):839–59. 10.1080/08959420.2021.1973341 34629015

[10] Clare L Wu Y-T Quinn C Jones IR Victor CR Nelis SM A comprehensive model of factors associated with capability to “live well” for family caregivers of people living with mild-to-moderate dementia: findings from the ideal study. Alzheimer Dis Assoc Disord. (2019) 33(1):29–35. 10.1097/WAD.0000000000000285 30802226 PMC6416095

[11] Age UK. Digital inclusion and older people – how have things changed in a Covid-19 world? London (2021).

